# Disparities in United States Nationwide County-Level PrEP Rate and PrEP-to-Need Ratio During the COVID-19 Pandemic

**DOI:** 10.1007/s40615-025-02429-2

**Published:** 2025-04-17

**Authors:** Sarah J. Miller, Amandeep Kaur, Xueying Yang

**Affiliations:** 1 Department of Psychology, University of South Carolina, Columbia, SC 29208, USA; 2 Arnold School of Public Health, South Carolina SmartState Center for Healthcare Quality, University of South Carolina, Columbia, SC 29208, USA; 3 Department of Epidemiology and Biostatistics, Arnold School of Public Health, University of South Carolina, Columbia, SC 29208, USA; 4 Department of Health Promotion, Education and Behavior, Arnold School of Public Health, University of South Carolina, Columbia, SC 29208, USA

**Keywords:** HIV prevention, PrEP, COVID-19, Disparities, Race

## Abstract

**Background:**

The COVID-19 pandemic created numerous barriers to pre-exposure prophylaxis (PrEP) access in the United States (US). The present study aimed to understand changes in county-level PrEP use relative to the need for PrEP during the COVID-19 pandemic, particularly for racial minorities.

**Method:**

Public county-level data from 2019 to 2022 were used. Linear mixed models with multiple imputations were used to examine changes in PrEP rate and PrEP-to-need ratio (PNR) across time, adjusting for covariates. We also assessed PNR and PrEP rate across time allowing for race and time interactions. Finally, we examined PNR and PrEP rate among age groups over time.

**Results:**

There was no difference in PrEP prevalence between 2019 and 2020 (*p* > 0.05). However, compared to 2019, PrEP prevalence was higher in 2021 and 2022 (*p* < .05). There was no change in PNR in 2020 or 2021 from 2019 (*p* > 0.05). Compared with 2019, PNR increased in 2022 (*p* < 0.05). Non-Hispanic Black population concentration did not predict the PrEP rate in 2020 or 2021 (*p* > 0.05), though it did predict a slight increase in PrEP prevalence in 2022 relative to 2019 (*p* < 0.05). Concentration non-Hispanic Black population did not predict PNR in any year (*p* > 0.05).

**Conclusion:**

Although PrEP use began increasing from pre-pandemic levels by 2021, PNR increases did not occur until 2022. Increases in PNR did not occur in areas with greater concentrations non-Hispanic Black individuals, suggesting disparities worsened during the pandemic. Addressing racial disparities is key in responding to COVID-19 related disruptions to HIV prevention efforts.

## Introduction

Pre-exposure prophylaxis (PrEP) is a medication that is highly effective in preventing HIV, with 99% effectiveness for men who have sex with men (MSM) and 74% effectiveness for people who inject drugs [1–4]. HIV prevention, including the use of PrEP, is a key pillar of the US ending the HIV epidemic (EHE) initiative, a federal plan focused on reducing the number of new HIV diagnoses in the US and improving HIV outcomes among those with HIV [5]. The COVID-19 pandemic has substantially disrupted routine medical care for many individuals, including PrEP users [8]. Research examining weekly-prescription filling of PrEP suggests a reduction in PrEP use in 2020 [6]. Longitudinal research between 2017 and 2021 suggests that the trend of increases in PrEP prescriptions stalled during the pandemic [7]. However, some clinics which implemented telehealth procedures found no reduction in PrEP prescriptions during 2020 [8]. Thus, existing research demonstrates mixed impacts of the COVID-19 pandemic on PrEP outcomes. Given that PrEP rates have increased since its approval in 2012, a reduction or lack of increase in PrEP prescriptions may represent stalled progress. While existing research demonstrates some effect of the COVID-19 pandemic on PrEP access, the extent to which these disruptions were sustained across time is unknown.

Social determinants of health (SDOH), including nonmedical factors such as economic stability, education access, healthcare access, neighborhood and built environment, and social and community access, are important predictors of PrEP access and uptake [9]. Thus, characteristics of where an individual lives can impact their ability to access PrEP. Those in areas with high poverty and income inequality have difficulty accessing PrEP [10, 11]. Likewise, those in rural areas are less likely to access or use PrEP [12, 13].

According to intersectionality theory, which is rooted in Black feminist theory, interlocking systems of oppression shape the experiences of individuals with healthcare systems and fuel the incidence of health-related disparities [14–17]. Individuals exist at the intersection of numerous identities (e.g., age, race, sexual identity) that shape their experiences and those with multiple marginalized identities frequently face adverse outcomes [14–17]. Indeed, racial disparities exist in PrEP use and corresponding HIV prevention benefits in the US. White MSM are more likely to access PrEP compared with Black MSM, despite Black MSM being at a greater risk for HIV [11, 18]. These disparities across the PrEP care continuum are fueled by structural racism and discrimination, including bias from healthcare providers, that fuel medical mistrust [19, 20]. Notably, when considering HIV prevention among various racial and ethnic groups, disparities are rooted in shared historical and current sociocultural perspectives. Thus, efforts to describe the impact of the COVID-19 pandemic on HIV prevention efforts must examine impacts based on intersectional identities.

While existing research has examined changes in PrEP prescriptions during the COVID-19 pandemic, relatively less is known about changes to PrEP prescriptions relative to the need in a geographic area. Certain areas, such as the US South and rural areas, have high prevalence rates of HIV, resulting in greater risk for HIV acquisition and corresponding higher needs for PrEP [21]. PrEP-to-need ratio (PNR) is a measure that aims to account for relative need for PrEP by examining the number of PrEP prescriptions in an area, divided by that area’s new diagnosis rate [22]. Thus, higher PNRs indicate that the need for PrEP prescriptions is met for more people.

While existing research suggests that there was a disruption in growth of PrEP use, but not a reduction in PrEP use itself, during the early months of the COVID-19 pandemic, the extent to which these changes lasted across time and their impact on racial disparities in PrEP access is currently unknown. Moreover, existing research has not accounted for the relative need for PrEP in given areas in changes in PrEP use during the COVID-19 pandemic. Therefore, the present study seeks to fill these gaps by examining annual national county-level PrEP and PNR rates between the years of 2019 and 2022. It also aims to understand the effects of the COVID-19 pandemic on PrEP-related racial disparities. Understanding the impact of the COVID-19 pandemic on HIV prevention efforts is critical to ensure current efforts address disparities that may have been exacerbated by the pandemic. Moreover, the findings from this study could also inform HIV prevention efforts in the midst of future public health crises.

## Methods

### Data Sources

Publicly available county-level data were used for the analyses from years 2019 to 2022 from AIDSVu, the American Community Survey (ACS), and the Centers for Disease Control and Prevention (CDC) social vulnerability index [22–24]. Because this study utilizes pre-existing publicly available county-level data without any personally identifiable information, institutional review board (IRB) approval was not obtained.

#### Outcome Variables

PrEP prevalence and PNR were obtained from AIDSVu [22]. PrEP prevalence is the number of PrEP prescriptions per 100,000 individuals in a county’s population. AIDSVu obtains PrEP data from IQVIA, which includes anonymized individual-level prescription records from pharmacies (i.e., retail pharmacies, traditional pharmacies, specialty mail-order pharmacies, long-term care facilities, other pharmacies) [22]. The data are compiled by researchers at Emory University [22]. PNR is the number of PrEP prescriptions divided by the number of new HIV diagnoses in a given county. A higher PNR is indicative of greater PrEP saturation. AIDSVu utilizes data from the CDC for the number of new HIV diagnoses in a given county [22]. Both PrEP prevalence and PNR were available by age subgroups. Additional information about the data can be found from AIDSVu [22].

#### Predictor Variables

Racial and ethnic composition of counties was used as a predictor. This data was obtained from the ACS 5-year estimates accessed from SocialExplorer.com [23]. Time, as defined by the year of outcome data (i.e., 2019, 2020, 2021, or 2022) was also included as a predictor variable. This was obtained from AIDSVu alongside the outcome variables.

#### Covariates

Based on prior literature and utilizing the theory of SDOH, SES vulnerability, income inequality, and county-level rurality were included as covariates [9]. These were included as covariates to ascertain the impact of the COVID-19 pandemic beyond these existing vulnerabilities based on social determinants of health.

Socio-economic status (SES) vulnerability was measured through the CDC’s social vulnerability index [25]. SES vulnerability was defined by the % living below the poverty line, the % unemployed, the income level, and the % with no high school diploma [25]. A higher score indicates greater socio-economic vulnerability in a county. Since SES data is collected every other year, for the current study we extracted data from 2018, assuming it remained constant in 2019. This was included as a covariate because prior research has demonstrated that SES and other economic variables predict access to PrEP [10, 11, 26].

The Gini coefficient for income inequality was obtained from ACS 5-year estimates accessed from SocialExplorer.com [23]. This measures inequality in the distribution of incomes within a county, with scores ranging from zero (perfect equality) to one (perfect inequality). This was included as a covariate because prior research has demonstrated income inequality to predict lower PNR [11].

County-level rurality was assessed using the National Center for Health Statistics’ 2013 Urban–Rural Classification scheme [24]. Data were coded such that 1 = large central metro, 2 = large fringe metro, 3 = medium metro, 4 = small metro, 5 = micropolitan, and 6 = non-core. This was included as a covariate because prior research has demonstrated lower access to PrEP in rural areas [12, 13].

### Statistical Analysis

For descriptive statistics, we conducted univariate analysis and reported mean and standard deviations for all study variables across study years (i.e., 2019–2022). For regression analysis, unadjusted and adjusted linear mixed models (LMM) were used. Adjusted models included covariates such as SES, county-level rurality, and Gini coefficient for income inequalities. First, we conducted LMM to assess PrEP and PNR, comparing rates from 2019 with 2020, 2021, and 2022. Next, LMM analyses were conducted for each age subgroup (i.e., aged < 25, 25–34, 35–44, 45–54, 55 + years old). Finally, LMM regressions were conducted to examine the PrEP prevalence and PNR by race, considering interaction of race (i.e., non-Hispanic White, non-Hispanic Black, Hispanic White, Hispanic Black) with year on PrEP prevalence and PNR. All analyses were conducted using SAS 9.4.

Given confidentiality concerns, AIDSVu suppresses data for counties with a low level of PrEP users and/or HIV cases, leading to missing value of certain variables in some counties [22]. We used multiple imputation to fill these missing data. Supplemental Tables 1–5 include a description of missing data for all variables. Multiple imputation procedure using “Proc MI” in SAS with five imputations were used to impute the missing information in the dataset. To overcome the challenges of outliers, which were not deleted to preserve that data and to ensure the validity of imputed data, we set a maximum value of PrEP users among age groups of 34–45, 45–54, and 55 and PNR among age group 55 to 9999 (or < 10,000). All regression analyses were conducted with multiple imputed data as well as using complete case analysis and results were compared.

## Results

Over the entire study period (2019–2022), the mean county PrEP prevalence was 69.34 PrEP users per 100,000 (SD = 95.82). The mean county PNR was 9.31 (SD = 9.03). Sample descriptives for all outcomes and predictors by year can be found in Table 1. Results of analyses utilizing multiple imputation are found in Table 2. Supplemental Table 6 includes results from the complete case analysis for all analyses, with areas of discrepancy between analytical approaches highlighted below.

### PrEP Prevalence

#### Changes in PrEP Prevalence Across Time

Table 2 demonstrated the temporal trend of PrEP prevalence over time. Overall, there was no difference in PrEP prevalence between 2019 and 2020 (*p* > 0.05). However, compared to 2019, PrEP prevalence was higher in 2021 (*B* = 10.02, SE = 3.61, *p* = 0.02), and 2022 (*B* = 19.33, SE = 4.16, *p* = 0.002). In contrast, using complete case analysis, PrEP prevalence was higher in 2020, 2021, and 2022 compared with 2019 (*p* < 0.05).

#### Changes in PrEP Prevalence Across Time by Age

Table 2 showed the change of PrEP prevalence over time by age group. Analysis of changes in PrEP prevalence across time by age category (i.e., < 25, 25–35, 35–44, 45–54, 55 +) revealed no changes for any ages between 2019 and 2020 or 2021 (*p* > 0.05). In 2022, compared with 2019, there was an increase for those ages < 25 years old (*B* = 17.72, SE = 6.42, *p* = 0.03) and those 35–44 years old (*B* = 30.67, SE = 9.39, *p* = 0.004), but not for any other age categories (*p* > 0.05). Using complete case analysis, PrEP prevalence was higher in 2020, 2021, and 2022 compared with 2019 for all age categories except those < 25 years old. For those < 25 years old, rates of PrEP prevalence were consistent between 2019 and 2020, before increasing in 2021 and 2022 compared with 2019 (*p* < 0.05).

#### Racial Disparities

Table 2 contains the results of LMM models predicting PrEP prevalence accounting for county-level racial distribution. At baseline (i.e., 2019), we observed that a greater percent non-Hispanic White individuals in the population was associated with lower PrEP prevalence (*B* = −0.80, SE = 0.09, *p* < 0.001). The composition of other races was not associated with PrEP prevalence. There was no change in PrEP prevalence between 2019 and 2020 regarding different racial composition. In 2021, compared with 2019, there was an increase in PrEP prevalence for each percentage point increase in non-Hispanic White population (*B* = 0.09, SE = 0.04, *p* = 0.04) and Hispanic Black population (*B* = 21.48, SE = 9.04, *p* = 0.02), but not for percent Hispanic White or non-Hispanic Black populations (*p* > 0.05). In 2022, compared with 2019, there was an increase in PrEP prevalence based on the percent non-Hispanic White population, non-Hispanic Black population, Hispanic White population, and Hispanic Black population (*p* < 0.05). In contrast, when using complete case analysis, there was a significant increase in 2020, 2021, and 2022 compared with 2019 based on non-Hispanic White population, non-Hispanic Black population, Hispanic White population, and Hispanic Black (*p* < 0.05).

### PNR

#### Changes in PNR Across Time

Table 3 showed the temporal trend of PNR across time. Overall, there was no change in PNR in 2020 or 2021 compared with 2019 (*p* > 0.05). However, PNR increased by 2.25 in 2022 (*B* = 2.25, SE = 0.97, *p* = 0.047) compared with 2019. In contrast, the complete case analysis indicated higher levels of PNR in 2020, 2021, and 2022 compared with 2019 (*p* < 0.05).

#### Changes in PNR Across Time by Age

Table 3 contains the results of changes of PNR over time by age group. For all age groups, there was no difference in PNR between 2020 and 2021 compared with 2019 (*p* > 0.05). In 2022, compared with 2019, there was an increase in PNR for those ages 35–44 (*p* < 0.05), but not for any other age groups (*p* > 0.05). However, using complete case analysis, PNR increased in 2020, 2021, and 2022 compared with 2019 for all age groups (*p* < 0.05).

#### Racial Disparities

Table 3 contains the results of LMM predicting PNR accounting for county-level racial distribution. At baseline (i.e., 2019), greater percentage of non-Hispanic White populations (*B* = −0.04, SE = 0.02, *p* = 0.03) and non-Hispanic Black population (*B* = −0.08, SE = 0.04, *p* = 0.04) were both associated with lower PNR. In 2020, 2021, and 2022, compared with 2019, there was no change in PNR based on percentage non-Hispanic White population, non-Hispanic Black population, Hispanic White population, or Hispanic Black population (*p* > 0.05). In contrast, using complete case analysis, there was an increase in PNR for each increase in non-Hispanic White population, non-Hispanic Black population, Hispanic White population, and Hispanic Black population in 2020, 2021, and 2022 compared with 2019 (*p* < 0.05).

## Discussion

To the best of our knowledge, this is one of the first studies examining the temporal trend of and the impact of COVID-19 pandemic on PrEP use and PNR across the US. Using nationwide PrEP data, this study has highlighted that, although PrEP prevalence was not significantly reduced and began rising in 2021, PNR did not increase from prepandemic levels until 2022. We also observed the racial disparities and age differences in PrEP use and PNR across years, with areas with populations with higher concentrations non-Hispanic Black and Hispanic White populations having slower increases in PrEP and PNR.

Although PrEP prevalence did not increase between 2019 and 2020, the present analyses indicated that compared with 2019, PrEP prevalence was higher in 2021 and 2022. Consistent with some prior research, there was not a reduction in overall PrEP prevalence during the COVID-19 pandemic [8]. Although some prior research is consistent with our findings, other research using weekly-prescription data finding a reduction in PrEP use in 2020 showed a reduction in PrEP use [6, 7]. This discrepancy in findings suggest that while changes may have occurred within 2020 in PrEP use, they were not so great to be reflected in the annual data for 2020. Furthermore, consistent with prior research, increases in PrEP prevalence did not occur until 2021 in the present study. This suggests that the barriers experienced by individuals during the COVID-19 pandemic may have stalled progress towards increases in PrEP prevalence. This stalled progress is important given that only 30% of those who could benefit from PrEP are currently prescribed it [18].

It is unlikely that changes in PrEP use were attributable solely to reductions in HIV-risk behaviors. Although qualitative research has suggested individuals may have reduced their sexual activity during the early phases of the COVID-19 pandemic, quantitative cross-sectional surveys have found no change or even increases in sexual partners following the start of the COVID-19 pandemic [28, 29]. Thus, while reductions in sexual behavior may have fueled some changes, these are likely not solely accounted for by changes in sexual behavior. This is consistent with the fact that PNR did not increase until 2022, suggesting that saturation of PrEP did not meet the relative need for PrEP.

Qualitative research with PrEP care providers can explain some of these findings. Care providers explained they experienced a reduction in demand for PrEP due to both changes of sexual behavior and priorities [30]. Care providers shared that shifting to telehealth created barriers to PrEP use including difficulty building rapport, difficulty connecting patients with resources, suspension of low-barrier services such as walk-in-appointments, and differential effects of telehealth on marginalized populations such as those who are unhoused [30]. In addition, care providers noted that PrEP providers frequently were used to manage the COVID-19 response and thus had competing priorities [30].

This study was the first, to the author’s knowledge, to examine the impact of the COVID-19 pandemic on PNR. Unlike PrEP prevalence which increased beginning in 2021, PNR did not significantly differ between 2019, 2020, and 2021. PNR did not increase until 2022. This suggests that even though PrEP prevalence increased in 2021, this increase was not proportionate to the relative need for PrEP based on HIV incidence, indicating the population with PrEP indicators grew faster than PrEP users. In the aftermath of the COVID-19 pandemic, these findings highlight the importance of effort placed on increasing uptake of PrEP.

Racial disparities were evident in changes in PrEP prevalence across time. While increases were seen for counties with higher non-Hispanic White populations in 2021, these increases were not seen for counties with higher non-Hispanic Black or Hispanic White populations until 2022. This is consistent with prior research finding PrEP disruptions to be greater for Black and Latinx populations [6]. While many providers pivoted to telehealth services, Black populations are less likely to access telehealth services than White populations, which may partially explain this disparity [8, 27, 31]. Racial disparities in changes in PrEP prevalence across time are particularly concerning given that those who had challenges accessing PrEP during the COVID-19 pandemic were more likely to seroconvert compared with their counterparts [32]. These findings highlight the importance of addressing racial disparities in efforts to increase PrEP uptake.

Subgroups based on age showed minimal changes in PrEP prevalence across years, likely due to limited power. For those under 25 and between the ages of 35–44, increases in PrEP prevalence did not occur until 2022. For those aged 25–34, 44–54, and over 55, no PrEP prevalence increases were observed during the study period. Likewise, minimal age effects were noted for PNR. For those under 25 and between the ages of 35–44, increases in PNR did not occur until 2022. For those aged 25–34, 44–54, and over 55, no PNR increases were observed during the study period. Prior research has shown those under 25 were more likely to discontinue PrEP use during the COVID-19 pandemic, which is consistent with the present findings that showed a slower increase in PrEP and PNR for those under 25 [8]. Although this age effect was found, the present findings likely reflect limited power and further analysis should examine age-specific impacts of the COVID-19 pandemic.

The present research has several limitations. First, a significant amount of data was missing due to either data suppression or other reasons from AIDSVu. While the present analyses handled this with multiple imputation, this high level of missing data likely introduced bias into the results. In addition, the assumption for multiple imputation of missing completely at random is not upheld given that data were suppressed and therefore not missing completely at random. It is worthwhile to explore other methods for handling missing data, such as IPW or synthetic data generation, in future studies to confirm the findings in the present study. In addition, data for SES were used from 2018 as a covariate, with the assumption SES remained relatively stable. However, it is possible that changes may have occurred during the study period. Finally, AIDSVu does not currently provide PrEP-related data by racial demographics for county-level data [22]. The present study used county-level racial composition to predict outcomes as a proxy for racial group PrEP use. However, future research should examine PrEP rates within specific racial subgroups to best understand the impact of the COVID-19 pandemic on PrEP uptake. Finally, although the present study aimed to account for sociocultural experiences by utilizing county-level race and ethnicity, other sociodemographic characteristics (e.g., nationality, immigration status, sexual and gender minority status) shape individuals’ experiences with healthcare access. While the present analyses represent the US as a whole, future research should examine the changes in PrEP use for additional subgroup populations based on their unique sociocultural identities and experiences.

Despite these limitations, the present research has several important implications for HIV prevention efforts in the present and future. The current research highlights that the COVID-19 pandemic temporarily stalled progress towards increased PrEP access and PNR. These findings are unsurprising given disruptions to healthcare systems during the pandemic. Although the acute phase of the COVID-19 pandemic is over, the present study has important implications for current and future efforts to address the HIV pandemic. Efforts are crucially needed to continue expanding access to PrEP, particularly in areas with the greatest need. While PrEP prescriptions began to rise in 2021, PNR did not rise until 2022, suggesting that the COVID-19 pandemic had a longer-lasting effect on areas with high need for PrEP compared with areas with lower need for PrEP. Efforts to rebuild PrEP uptake, particularly among those populations at greatest risk for HIV, are crucially needed.

Beyond the impact of the COVID-19 pandemic, the present study demonstrates the importance of attending to HIV prevention efforts in the midst of other public health crises, particularly those related to infectious diseases. Although increases in PrEP and PNR have returned, the stalled progress affected communities at highest risk for HIV at high rates. Using lessons from the COVID-19 pandemic, in future public health crises, intentional effort must be placed on ensuring HIV prevention efforts—including PrEP—are prioritized, particularly in areas with high levels of HIV.

## Supplementary Material

Supplementary materials

**Supplementary Information** The online version contains supplementary material available at https://doi.org/10.1007/s40615-025-02429-2.

## Figures and Tables

**Table 1:** Descriptive statistics for all study variables across study period

Variable	Overall M (SD) [Range]	2019 M (SD) [Range]	2020 M (SD) [Range]	2021 M (SD) [Range]	2022 M (SD) [Range]

PrEP Rate	69.34 (95.82) [0 – 2446]	55.36 (108.37) [0 – 2446]	59.49 (95.57) [0–2056]	71.80 (86.54) [0 – 1676)	86.11 (94.55) [0 – 1852]
PrEP Rate Ages <25		70.55 (122.58) [0 – 2099]	69.91 (75.45) [0 – 1189]	84.55 (59.17) [0–695.00]	98.50 (59.99) [0–573]
PrEP Rate Ages 25–34		181.76 (410.31) [0 – 10524]	191.33 (318.28) [0–8082]	223.12 (250.98) [0–6142]	263.64 (274.34) [0–7133]
PrEP Rate Ages 35–44		124.32 (353.08) [0 – 7107]	129.59 (281.49) [0 – 5619]	84.55 (59.17) [0–695]	178.68 (300.22) [0–6739]
PrEP Rate Ages 45–54		77.05 (171.60) [0 – 2250]	78.53 (153.72) [0–1986]	88.19 (155.14) [0–2119]	94.74 (122.32) [0–1479]
PrEP Rate Ages 55+		33.19 (69.58) [0 = 942]	37.56 (71.29) [0–1019]	41.70 (61.25) [0–821]	47.78 (59.70) [0–911]
PNR	9.31 (9.03) [0.46 – 90.40]	6.30 (5.77) [0.46–39.85]	5.80 (7.69) [0.55–52.80]	10.27 (9.24) [0.73 – 70.40]	12.74 (11.36) [0.86–90.40]
PNR Ages < 25	7.79 (10.08) [0.50 – 128.00]	5.85 (8.87) [0.50 – 81.00]	6.82 (8.14) [0.55 – 76.50]	8.41 (9.90) [0.50 – 98.00]	10.02 (12.34) [0.56–128.00]
PNR Ages 25–34	10.44 (11.16) [0.60–142.00]	7.20 (6.93) [0.60–50.00]	9.42 (9.86) [0.60 – 87.00]	11.50 (11.77) [0.86 – 116.67]	13.91 (14.02) [1.13 – 142.00]
PNR Ages 35–44	12.36 (12.88) [0.71 – 117.00]	7.80 (6.53) [0.71 – 58.50]	11.08 (10.94) [0.71 – 76.67)	13.88 (13.20) [0.87 – 88.35]	17.26 (16.66) [1.17 – 117.00]
PNR Ages 45–54	10.96 (9.92) [0.54 – 68.00]	7.12 (43.56) [0.54 – 44.10]	10.67 (9.22) [0.89 – 54.25]	12.25 (10.03) [1.18 – 57.41]	14.34 (11.97) [1.00 – 68.00]
PNR Ages 55+	12.86 (13.87) [0.86 – 106.50]	6.87 (6.35) [1.13 –44.33]	12.10 (12.79) [0.86 – 82.50]	14.62 (14.19) [1.20 – 93.75]	18.33 (17.52) [1.00 – 106.50]
Percent Non Hispanic White	77.26 (19.89)	76.25 (20.22)	75.75 (20.22)	75.19 (20.26)	
Percent Non Hispanic Black	8.39 (14.41)	8.93 (14.43)	8.88 (14.37)	8.80 (14.31)	
Percent Hispanic White	5.66 (10.38)	6.67 (11.37)	6.00 (10.13)	5.21 (8.85)	
Percent Hispanic Black	0.11 (0.24)	0.15 (0.31)	0.14 (0.30)	0.14 (0.28)	

**Table 2:** Results of Mixed Linear Regression Models Predicting PrEP with Multiple Imputations

Model	Predictor	Estimate	Standard Error	DF	t Value	*p*

Adjusted Model: PrEP Prevalence	2019	0				
	2020	2.93	3.92	8.90	0.75	0.47
	2021	10.02	3.61	10.69	2.77	0.02*
	2022	19.33	4.16	8.00	4.64	0.002*

Adjusted Model: PrEP Prevalence Ages < 25	2019	0				
	2020	2.79	8.26	5.67	0.34	0.75
	2021	9.55	6.76	6.90	1.41	0.20
	2022	17.72	6.42	7.40	2.76	0.03*

Adjusted Model: PrEP Prevalence Ages 25–34	2019	0				
	2020	4.76	14.94	9.05	0.32	0.76
	2021	20.13	14.98	5.52	1.34	0.21
	2022	39.83	18.69	6.48	2.13	0.07

Adjusted Model: PrEP Prevalence Ages 35–44	2019	0				
	2020	5.47	14.60	6.63	0.37	0.72
	2021	16.93	11.36	10.02	1.49	0.17
	2022	30.67	9.39	18.79	3.26	0.004*

Adjusted Model: PrEP Prevalence Ages 45–54	2019	0				
	2020	−10.86	9.35	194.51	−1.16	0.25
	2021	−10.52	14.15	10.20	−0.74	0.47
	2022	−9.88	13.09	12.63	−0.76	0.46

Adjusted Model: PrEP Prevalence Ages 55+	2019	0				
	2020	−1.42	4.60	207.03	−0.31	0.76
	2021	5.29	5.42	27.56	0.97	0.34
	2022	7.06	4.98	55.88	1.42	0.16

Adjusted Model: PrEP Prevalence with Non-Hispanic White	Non-Hispanic White	−0.80	0.09	314.8	−8.52	<.0001
	Non-Hispanic White * 2020	0.02	0.04	16.16	0.35	0.73
	Non-Hispanic White * 2021	0.09	0.04	28.90	2.24	0.04*

	Non-Hispanic White * 2022	0.19	0.04	15.01	4.24	0.0007*

Adjusted Model: PrEP Prevalence with Non-Hispanic Black	Non-Hispanic Black	−0.21	0.19	18.51	−1.08	0.29

	Non-Hispanic Black * 2020	0.02	0.02	15.62	0.31	0.76

	Non-Hispanic Black * 2021	0.32	0.19	23.59	1.70	0.10

	Non-Hispanic Black * 2022	0.59	0.23	9.41	2.63	0.03*

Adjusted Model: PrEP Prevalence with Hispanic White	Hispanic White	−0.15	0.23	19.64	−0.66	0.52

	Hispanic White * 2020	0.20	0.27	16.73	0.74	0.47

	Hispanic White * 2021	0.51	0.33	13.55	1.57	0.14

	Hispanic White * 2022	0.97	0.30	13.55	3.20	0.007*

Adjusted Model: PrEP Prevalence with Hispanic Black	Hispanic Black	−7.40	9.14	12.59	−0.81	0.43

	Hispanic Black * 2020	7.98	9.69	19.94	0.82	0.42

	Hispanic Black * 2021	21.48	9.04	38.67	2.38	0.02*

	Hispanic Black * 2022	21.95	7.44	45.16	2.95	0.005*

* p < .05

**Table 3:** Results of Mixed Linear Regression Models Predicting PNR with Multiple Imputations

Model	Predictor	Estimate	Standard Error	DF	t Value	*p*

Adjusted Model: PNR	2019	0				
	2020	0.38	0.94	9.58	0.40	0.70
	2021	1.00	0.83	13.33	1.20	0.25
	2022	2.25	0.97	8.89	2.31	0.05

Adjusted Model: PNR Ages < 25	2019	0				
	2020	0.78	1.65	6.42	0.47	0.65
	2021	1.21	1.65	6.41	0.74	0.49
	2022	2.46	1.31	8.98	1.87	0.09

Adjusted Model: PNR Ages 25–34	2019	0				
	2020	0.09	1.00	11.55	0.09	0.93
	2021	0.59	0.99	11.91	0.60	0.56
	2022	1.71	1.34	6.74	1.28	0.24

Adjusted Model: PNR Ages 35–44	2019	0				
	2020	0.63	1.52	6.36	0.42	0.69
	2021	1.29	1.27	8.07	1.01	0.34
	2022	2.93	1.10	10.96	2.66	0.02*

Adjusted Model: PNR Ages 45–54	2019	0				
	2020	0.39	0.75	75.24	0.52	0.61
	2021	0.87	0.79	43.03	1.11	0.27
	2022	1.30	0.84	27.26	1.55	0.13

Adjusted Model: PNR Ages 55+	2019	0				
	2020	4.31	14.47	21.15	0.30	0.77
	2021	14.85	14.94	27.58	0.99	0.33
	2022	11.49	15.70	14.78	0.73	0.48

Adjusted Model: PNR with Non-Hispanic White	Non-Hispanic White	−0.04	0.02	89.13	−2.26	0.03*

	Non-Hispanic White * 2020	−0.00	0.02	7.02	−0.08	0.94

	Non-Hispanic White * 2021	0.01	0.02	7.89	0.53	0.61

	Non-Hispanic White * 2022	0.02	0.02	6.60	1.15	0.29

Adjusted Model: PNR with Non-Hispanic Black	Non-Hispanic Black	−0.08	0.04	42.33	−2.13	0.04*

	Non-Hispanic Black * 2020	0.01	0.04	167.65	0.19	0.85

	Non-Hispanic Black * 2021	0.03	0.05	70.67	0.68	0.50

	Non-Hispanic Black * 2022	0.07	0.04	51.93	1.57	0.12

Adjusted Model: PNR with Hispanic White	Hispanic White	0.00	0.05	28.19	0.10	0.91

	Hispanic White * 2020	0.02	0.08	12.24	0.21	0.84

	Hispanic White * 2021	0.02	0.09	12.69	0.20	0.85

	Hispanic White * 2022	0.07	0.08	19.38	0.94	0.36

Adjusted Model: PNR with Hispanic Black	Hispanic Black	−1.60	1.77	58.13	−0.90	0.37

	Hispanic Black * 2020	1.23	2.41	40.78	0.51	0.61

	Hispanic Black * 2021	2.23	2.23	186.56	1.00	0.32

	Hispanic Black * 2022	2.70	1.89	62.96	1.43	0.16

* p < .05

## Data Availability

All data utilized in this study are available for public access and use. PrEP and PNR data can be found from AIDSVu’s Website (https://aidsvu.org/resources/#/datasets, Accessed in May 2023). The American Community Survey, which provided racial and ethnic compositions of counties and the Gini coefficient for income inequality, can be found on SocialExplorer.com (https://www.socialexplorer.com/home/, Accessed in July 2023). SES Vulnerability can be found in the Centers for Disease Control and Prevention’s Social Vulnerability Index Data (https://www.atsdr.cdc.gov/placehealth/php/svi/index.html), Accessed in September 2022). The Center for Health Statistics’ 2013 Urban-Rural Classification scheme is available through the Centers for Disease Control and Prevention (https://www.cdc.gov/nchs/data_access/urban_rural.htm, Accessed in October 2022).
