# Supplementary material for: Disparities in United States Nationwide County-Level PrEP Rate and PrEP to Need Ratio During the COVID-19 Pandemic
Source: J Racial Ethn Health Disparities. 2025 Apr 17;13(3):2428–37. doi: 10.1007/s40615-025-02429-2 (PMC12917910; doi:10.1007/s40615-025-02429-2)
Supplement: Supplementary file 1 — Supplementary file1 (DOCX 32 KB) [file 40615_2025_2429_MOESM1_ESM.docx]

**Disparities in United States Nationwide County-Level PrEP Rate and PrEP to Need Ratio during the COVID-19 Pandemic**

Journal of Ethnic and Racial Health Disparities

**Authors**

Sarah J. Miller^1,2*^

Amandeep Kaur^3^

Xueying Yang^2,4^

1 Department of Psychology, University of South Carolina, Columbia, SC, USA 29208

2 South Carolina SmartState Center for Healthcare Quality, Arnold School of Public Health, University of South Carolina, Columbia, SC, USA 292083

3 Department of Epidemiology and Biostatistics, Arnold School of Public Health, University of South Carolina, SC, USA, 29208

4 Department of Health Promotion, Education and Behavior, Arnold School of Public Health, University of South Carolina, Columbia, SC, USA, 29208

***Correspondence:**

Correspondence related to this article can be sent to Sarah J Miller at [sjm8@email.sc.edu](mailto:sjm8@email.sc.edu).

Supplemental Table 1: Overall Proportion of Missingness

|  | **N (%)** |
| --- | --- |
| Missing | 150 (4.77) |
| Not missing | 2993 (95.23) |

Supplemental Table 2: Proportion of missingness for PrEP Rate and PNR

|  | |
| --- | --- |
|  | **N (%)** |
| PrEP Rate | |
| Not missing | 1751 (55.71) |
| Missing | 1392 (44.29) |
| PNR | |
| Not missing | 708 (22.55) |
| Missing | 2432 (77.45) |

Supplemental Table 3: Number of Missingness for Demographics, and Different Age Groups for PrEP Rate and PNR

| **Variable** | **N** | **N Miss** |
| --- | --- | --- |
| PrEP Rate < 24 years  PrEP Rate 25-34 years  PrEP Rate 35-44 years  PrEP Rate 45-54 years  PrEP Rate 55+ years  PNR < 24 years  PNR 25-34 years  PNR 35-44 years  PNR 45-54 years  PNR 55+ years  Non-Hispanic White  Non-Hispanic Black  Hispanic White  Hispanic Black  Non-Hispanic White  Non-Hispanic Black  Non-Hispanic American Indian  Non-Hispanic Asian  Non-Hispanic Native Hawaiian  Non-Hispanic Other  Non-Hispanic Two or More  Hispanic White  Hispanic Black  Hispanic American Indian  Hispanic Asian  Hispanic Native Hawaiian  Hispanic Other  Hispanic Two or More  Rural  Gini Coefficient  SES Vulnerability  Time | 581  1047  714  578  462  236  359  239  184  158  3141  3141  3141  3141  3141  3141  3141  3141  3141  3141  3141  3141  3141  3141  3141  3141  3141  3141  3141  3141  3140  3143 | 2562  2096  2429  2565  2681  2907  2784  2904  2959  2985  2  2  2  2  2  2  2  2  2  2  2  2  2  2  2  2  2  2  2  2  3  0 |

Supplemental Table 4: Missing Data for Model 1 – PrEP Rate Outcome

| **Variables** | **N** | **N Miss** |
| --- | --- | --- |
| PrEP Rate  Non-Hispanic White  Non-Hispanic Black  Hispanic White  Hispanic Black  Non-Hispanic White  Non-Hispanic Black  Non-Hispanic American Indian  Non-Hispanic Asian  Non-Hispanic Native Hawaiian  Non-Hispanic Other  Non-Hispanic Two or More race  Hispanic White  Hispanic Black  Hispanic American Indian  Hispanic Asian  Hispanic Native Hawaiian  Hispanic Other  Hispanic Two or More race  Rural  Gini Coefficient  SES Vulnerability  Time | 1751  3141  3141  3141  3141  3141  3141  3141  3141  3141  3141  3141  3141  3141  3141  3141  3141  3141  3141  3141  3141  3140  3143 | 1392  2  2  2  2  2  2  2  2  2  2  2  2  2  2  2  2  2  2  2  2  3  0 |

Supplemental Table 5: Relationship Between Missing Data and Observed Data Odds Ratio Estimates

| **Effect** | **Point Estimate** | **95% Wald Confidence Limits** | |
| --- | --- | --- | --- |
| Rural* | 14.714 | 10.258 | 21.105 |
| Gini Coefficient* | 0.207 | 0.056 | 0.773 |
| SES Vulnerability | 1.034 | 0.915 | 1.169 |
| Non-Hispanic White* | 0.389 | 0.280 | 0.541 |
| Non-Hispanic Black* | 0.909 | 0.853 | 0.968 |
| Non-Hispanic American Indian | 1.012 | 0.964 | 1.062 |
| Non-Hispanic Asian* | 0.824 | 0.774 | 0.877 |
| Non-Hispanic Native Hawaiian* | 0.926 | 0.886 | 0.967 |
| Non-Hispanic Other* | 0.820 | 0.786 | 0.856 |
| Non-Hispanic Two or More* | 1.155 | 1.050 | 1.271 |
| Hispanic White | 1.057 | 0.971 | 1.150 |
| Hispanic Black* | 0.838 | 0.802 | 0.876 |
| Hispanic American Indian | 1.005 | 0.961 | 1.051 |
| Hispanic Asian* | 0.876 | 0.824 | 0.931 |
| Hispanic Native Hawaiian* | 0.884 | 0.815 | 0.958 |
| Hispanic Other | 0.968 | 0.916 | 1.024 |
| Hispanic Two or More* | 0.890 | 0.846 | 0.937 |

*Statistically significant at p-value<0.05

Supplemental Table 6: Results of mixed model regression models using complete case analysis

| **Model** | **Predictor** | **Estimate** | **Standard Error** | **DF** | **t Value** | ***p*** |
| --- | --- | --- | --- | --- | --- | --- |
| Adjusted Model: PrEP Prevalence | 2019 | 0 |  |  |  |  |
|  | 2020 | 5.37 | 0.69 | 5589 | 7.83 | <.0001** |
|  | 2021 | 21.80 | 0.68 | 5589 | 32.26 | <.0001** |
|  | 2022 | 39.03 | 0.68 | 5589 | 57.79 | <.0001** |
| Adjusted Model: PrEP Prevalence with Non-Hispanic White | NH White | -1.09 | 0.12 | 3537 | -9.25 | <.0001** |
|  | NH White * 2019 | 0 |  |  |  |  |
|  | NH White * 2020 | 0.05 | 0.01 | 3537 | 5.86 | <.0001** |
|  | NH White * 2021 | 0.24 | 0.01 | 3537 | 26.05 | <.0001** |
| Adjusted Model: PrEP Prevalence with Non-Hispanic Black | NH Black | -0.37 | 0.16 | 3537 | -2.23 | 0.0256* |
|  | NH Black * 2019 | 0 |  |  |  |  |
|  | NH Black * 2020 | 0.24 | 0.04 | 3537 | 5.65 | <.0001** |
|  | NH Black * 2021 | 0.91 | 0.04 | 3537 | 21.44 | <.0001** |
| Adjusted Model: PrEP Prevalence with Hispanic White | HispWhite | -0.41 | 0.18 | 3537 | -2.30 | 0.0214* |
|  | HispWhite * 2019 | 0 |  |  |  |  |
|  | HispWhite * 2020 | 0.44 | 0.07 | 3537 | 6.70 | <.0001** |
|  | HispWhite * 2021 | 1.54 | 0.09 | 3537 | 18.01 | <.0001** |
| Adjusted Model: PrEP Prevalence with Hispanic Black | HispBlack | -18.39 | 3.62 | 3537 | -5.08 | <.0001** |
|  | HispBlack * 2019 | 0 |  |  |  |  |
|  | HispBlack * 2020 | 14.46 | 2.01 | 3537 | 7.20 | <.0001** |
|  | HispBlack * 2021 | 43.07 | 2.04 | 3537 | 21.07 | <.0001** |
| Adjusted Model: PrEP Prevalence Ages < 25 | 2019 | 0 |  |  |  |  |
|  | 2020 | 1.74 | 1.76 | 1797 | 0.99 | 0.3231 |
|  | 2021 | 19.59 | 1.71 | 1797 | 11.44 | <.0001** |
|  | 2022 | 37.83 | 1.69 | 1797 | 22.38 | <.0001** |
| Adjusted Model: PrEP Prevalence Ages 25-34 | 2019 | 0 |  |  |  |  |
|  | 2020 | 11.58 | 3.52 | 3419 | 3.29 | 0.001* |
|  | 2021 | 56.72 | 3.45 | 3419 | 16.44 | <.0001** |
|  | 2022 | 107.40 | 3.44 | 3419 | 31.23 | <.0001** |
| Adjusted Model: PrEP Prevalence Ages 35-44 | 2019 | 0 |  |  |  |  |
|  | 2020 | 10.34 | 3.43 | 2374 | 3.02 | 0.0026* |
|  | 2021 | 40.32 | 3.36 | 2374 | 12.00 | <.0001** |
|  | 2022 | 80.59 | 3.34 | 2374 | 24.10 | <.0001** |
| Adjusted Model: PrEP Prevalence Ages 45-54 | 2019 | 0 |  |  |  |  |
|  | 2020 | 3.65 | 1.80 | 1816 | 2.03 | 0.0427* |
|  | 2021 | 16.00 | 1.78 | 1816 | 9.00 | <.0001** |
|  | 2022 | 27.15 | 1.76 | 1816 | 15.41 | <.0001** |
| Adjusted Model: PrEP Prevalence Ages 55+ | 2019 | 0 |  |  |  |  |
|  | 2020 | 6.32 | 0.95 | 1520 | 6.62 | <.0001* |
|  | 2021 | 14.32 | 0.94 | 1520 | 15.28 | <.0001* |
|  | 2022 | 24.39 | 0.93 | 1520 | 26.12 | <.0001* |
| Adjusted Model: PNR | 2019 | 0 |  |  |  |  |
|  | 2020 | 2.1409 | 0.1905 | 1844 | 11.24 | <.0001* |
|  | 2021 | 4.3724 | 0.1900 | 1844 | 23.01 | <.0001* |
|  | 2022 | 6.8693 | 0.1900 | 1844 | 36.16 | <.0001* |
| Adjusted Model: PNR with Non-Hispanic White | NH White | -0.025 | 0.014 | 1198 | -1.83 | 0.0671 |
|  | NH White * 2019 | 0 |  |  |  |  |
|  | NH White * 2020 | 0.03 | 0.003 | 1198 | 12.00 | <.0001* |
|  | NH White * 2021 | 0.07 | 0.003 | 1198 | 25.20 | <.0001* |
| Adjusted Model: PNR with Non-Hispanic Black | NH Black | -0.15 | 0.02 | 1198 | -9.25 | <.0001* |
|  | NH Black * 2019 | 0 |  |  |  |  |
|  | NH Black * 2020 | 0.05 | 0.01 | 1198 | 5.03 | <.0001* |
|  | NH Black * 2021 | 0.09 | 0.01 | 1198 | 10.44 | <.0001* |
| Adjusted Model: PNR with Hispanic White | HispWhite | -0.03 | 0.02 | 1198 | -1.79 | 0.0731 |
|  | HispWhite * 2019 | 0 |  |  |  |  |
|  | HispWhite * 2020 | 0.11 | 0.02 | 1198 | 7.77 | <.0001* |
|  | HispWhite * 2021 | 0.26 | 0.02 | 1198 | 15.34 | <.0001* |
| Adjusted Model: PNR with Hispanic Black | HispBlack | -1.92 | 0.55 | 1198 | -3.47 | 0.005* |
|  | HispBlack * 2019 | 0 |  |  |  |  |
|  | HispBlack * 2020 | 2.77 | 0.37 | 1198 | 7.53 | <.0001* |
|  | HispBlack * 2021 | 5.36 | 0.38 | 1198 | 14.28 | <.0001* |
| Adjusted Model: PNR Ages < 25 | 2019 | 0 |  |  |  |  |
|  | 2020 | 1.21 | 0.43 | 637 | 2.79 | 0.0054* |
|  | 2021 | 3.07 | 0.43 | 637 | 7.15 | <.0001* |
|  | 2022 | 4.85 | 0.43 | 637 | 11.36 | <.0001* |
| Adjusted Model: PNR Ages 25-34 | 2019 |  |  |  |  |  |
|  | 2020 | 2.39 | 0.35 | 932 | 6.73 | <.0001* |
|  | 2021 | 4.64 | 0.35 | 932 | 13.09 | <.0001* |
|  | 2022 | 7.04 | 0.35 | 932 | 19.90 | <.0001* |
| Adjusted Model: PNR Ages 35-44 | 2019 | 0 |  |  |  |  |
|  | 2020 | 3.62 | 0.57 | 606 | 6.37 | <.0001* |
|  | 2021 | 6.48 | 0.57 | 606 | 11.39 | <.0001* |
|  | 2022 | 9.86 | 0.57 | 606 | 17.34 | <.0001* |
| Adjusted Model: PNR Ages 45-54 | 2019 | 0 |  |  |  |  |
|  | 2020 | 3.08 | 0.48 | 459 | 6.42 | <.0001* |
|  | 2021 | 4.65 | 0.48 | 459 | 9.71 | <.0001* |
|  | 2022 | 6.74 | 0.48 | 459 | 14.07 | <.0001* |
| Adjusted Model: PNR Ages 55+ | 2019 | 0 |  |  |  |  |
|  | 2020 | 4.76 | 0.78 | 414 | 6.11 | <.0001* |
|  | 2021 | 7.47 | 0.78 | 414 | 9.62 | <.0001* |
|  | 2022 | 11.20 | 0.78 | 414 | 14.42 | <.0001* |

* p < .05
